# A Retrospective, Longitudinal External Study of the robustness and reproducibility of National Antibacterial Prescribing Survey Data

**DOI:** 10.1007/s11096-022-01411-w

**Published:** 2022-06-06

**Authors:** Zahraa Mahdi Abbas, Jeff Hughes, Bruce Sunderland, Petra Czarniak

**Affiliations:** Pharmacy, School of Medicine, Faculty of Health Sciences, Curtin University, Kent St, 6102 Bentley, Western Australia Australia

**Keywords:** Anti-infective agents, Antibacterial Agents, Antimicrobial stewardship, Australia, Bacterial drug resistance, Reproducibility of results

## Abstract

**Background:**

Point prevalence surveys are used internationally to audit antibacterial use as well as the impact of interventions on improving prescribing and resistance rates. The annual National Antibacterial Prescribing Survey provides data on the appropriateness of antibacterial agent prescribing in Australian hospitals. Assessing the survey’s robustness and result reproducibility is essential to its role in improving antibacterial prescribing practice.

**Aim:**

To evaluate the reproducibility of internal assessments of antibacterial agent prescribing of both guideline compliance and appropriateness from a Western Australian hospital.

**Method:**

Census data of 1051 prescriptions from 2013 to 2017 surveys were independently assessed for compliance based on Australian Therapeutic Guidelines - Antibiotics, and appropriateness, based on agent selection, therapy duration and microbiological test results. Concordance of these findings with internal hospital assessments was analysed.

**Results:**

This external study did not reproduce internal hospital audit results for compliance with guideline parameters. Non-compliant prescribing rate was significantly (p < 0.001) higher externally at 50.7% (533/1051) than internal assessment at 34.9% (367/1051). External analysis also found a significantly smaller proportion of prescriptions to be appropriate (551/1051, 52.4%) compared to internal analysis (745/1051, 70.9%) p < 0.001. Cohen’s Kappa analysis found a moderate agreement for compliance (0.49) and appropriateness (0.50) between the external and internal evaluations.

**Conclusion:**

The lack of adequate reproducibility of compliance and appropriateness assessments may limit the generalisability of the audit’s results. Validating point prevalence surveys that assess antibacterial agent prescribing can increase confidence and improve reproducibility of their findings; as they provide important data for antimicrobial stewardship programs.

**Supplementary information:**

The online version contains supplementary material available at 10.1007/s11096-022-01411-w.

## Impact statements

- To reduce the risk of antimicrobial resistance, hospital antibacterial prescribing should be optimal and based on evidence-based guidelines.
- Audit tools, such as the National Antimicrobial Prescribing Survey (NAPS) in Australia, are useful to monitor antimicrobial stewardship.
- To minimise ambiguity of the rationale for prescribed antibiotics and promote reproducibility, audit tools should align with national antibiotic prescribing guidelines.
- Implementation of a commentary section in the NAPS data collection form, to outline reasons for assessment decisions not aligned with guidelines, could improve their reproducibility and impact on improving antimicrobial stewardship.

## Introduction

Increased global prevalence of antibacterial resistance (AR) due to inappropriate antibacterial agent prescribing and usage has significantly increased rates and average length of hospitalisation, patient morbidity, mortality and associated societal and healthcare costs [1–5]. Antimicrobial Stewardship (AMS) aims to improve antimicrobial agent use to optimise patient outcomes and reduce adverse effects and AR. As part of AMS, point prevalence surveys (PPS) may be used for assessing the quality of antimicrobial use in the hospital setting, and to inform interventions for its improvement. Repetition of validated PPS offers a reliable overview of trends and the impact of applied interventions on antimicrobial use [6–9].

Different countries utilise PPS to evaluate and report on the appropriateness of their antimicrobial consumption [10, 11]. A PPS of Australian adult hospital patients reported an inappropriate antibacterial prescribing rate of 47% [12]. Similarly, a Chinese study found 51.4% of prescriptions inappropriate [13] and in American hospitals, up to 50% of antibiotic prescribing was incorrect due to therapy duration, indication or agent choice [14]. In Australia, the National Antimicrobial Prescribing Survey (NAPS), commenced in 2011, and its AMS audit tool has been increasingly used. In 2017, NAPS was undertaken by 314 Australian public and private hospitals, and comprised an analysis of 26,277 antimicrobial prescriptions [15]. Educational tools, online modules and case examples are provided to assist auditors in the completion of the PPS. However, NAPS acknowledges the limitation that these assessments are subjective and dependent upon interpretation by hospital personnel [15]. Assessment of the compliance parameter for the NAPS annual audit is based on the Australian Therapeutic Guidelines - Antibiotics [16], except where local and hospital based guidelines apply. Alternatively, therapy may be directed by infectious diseases clinicians or microbiological test results. Where therapy is based on a diagnosed indication, agent selection, dose, route of administration as well as duration of therapy are assessed for compliance with guidelines for that indication. A separate assessment for appropriateness also considers parameters such as a patient’s medical history, local resistance patterns and medication supply issues; which may introduce subjectivity and raise the ambiguity of inter- and intra-rater reliability and result reproducibility.

Concerns regarding scientific data integrity and reproducibility have received increased international interest [17, 18]. Reportedly up to 94% of medical study results are not entirely reproducible, with 65% reporting inconsistent results [18, 19]. To improve reproducibility, a robust study protocol is required that can standardise the collection and analysis of data variables across replicate studies [19, 20]. The NAPS has thus far not been subjected to an external audit process.

### Aim

This study aimed to externally validate the level of compliance with guidelines and additionally appropriateness of antibacterial agent prescribing at a large teaching hospital in Western Australia (WA). Concordance between the internal and external evaluations and data reproducibility were also evaluated.

### Ethics approval

The WA Health Governance, Evidence, Knowledge and Outcome system (GEKO) reviewed and approved this study as a Quality Improvement Activity (study number 18,599). Reciprocal ethics approval was provided by Curtin University Human Research Ethics Committee (HRE2017-0843; date approved: 05/12/2017).

## Method

### Study design

A retrospective, repeated cross-sectional, observational study analysed hospital NAPS; pre-collected in November of each year from 2013 to 2016, and in October of 2017. The data sets included all antibacterial agents prescribed at 8:00am on the day of the survey, as well as any surgical prophylaxis or single doses administered in the previous 24 h. Data were collected by hospital pharmacists alongside infectious diseases clinicians at a 450 bed adult teaching hospital in WA.

### Data collection

Raw NAPS data, collected on NAPS data collection forms (supplementary material) [15] were obtained as Excel sheets and combined into a single dataset before applying data exclusions; only agents administered via the parenteral, oral and intraperitoneal routes were included; topical, inhaled and vaginal antibacterials, as well as antifungals and antivirals were excluded. Duplicate entries were removed.

### Data analysis

Compliance was assessed using the Australian Therapeutic Guidelines - Antibiotic (Version 14 (2010) [21] for 2013 and 2014 data; Version 15 (2015) [16] for 2015–2017), whilst prescribing appropriateness was assessed based on NAPS guidelines (supplementary material). Compliance was based on the recorded indication for prescribing, agent selection, dosage, frequency, route of administration and duration of therapy. Each record was then given an overall classification of “Compliant”, “Not compliant”, “Directed therapy”, “No guidelines available” or “Not assessable”. Prescribing appropriateness was categorised as “Appropriate”, “Inappropriate”, and “Not assessable”, based on appropriate agent selection for the identified or likely causative organism, therapy duration, agent spectrum of action, and where available based on microbiological tests.

The Western Australian Therapeutic Advisory Group (WA-TAG) Surgical Antibiotic Prophylaxis Guideline: Adults [22], Australian Medicines Handbook [23] and hospital specific treatment guidelines were consulted where relevant. The external data analysis was performed initially by one clinical pharmacist. Where any doubt existed, a multidisciplinary discussion included three academic pharmacists all with clinical and research experience in infectious diseases and antimicrobial stewardship.

The data set was imported into SPSS Statistics 25 software for statistical analysis (IBM Corp. Armonk NY). The level of agreement between internal and external assessments with respect to compliance and appropriateness parameters were assessed using Cohen’s kappa statistic. The one-sample Chi-square statistic was used to assess whether the numbers of prescriptions observed within the 5 categories of compliance for the external assessment were similar to the distribution across these categories observed in the internal assessment. A similar analysis was performed for appropriateness. Intra-rater reliability (for the external assessment) was optimised by a single researcher utilising the same tools and methods throughout assessment; and finally an independent random cross-check of 30 assessments of guideline compliance and appropriateness by the three pharmacist members of the academic research team produced identical results.

A p-value < 0.05 indicated a statistically significant association for the Chi-square tests. The interpretation of Cohen’s kappa statistic was as follows: <0.40 indicated “poor to fair agreement”; 0.40–0.60 “moderate agreement”; 0.60–0.80 “substantial agreement” and > 0.80 “excellent agreement” [24].

## Results

Of 1518 antimicrobial prescriptions, 350 records were excluded (107 topical, inhaled and vaginal; 111 antivirals, 128 antifungals, and four duplicate entries), leaving 1168 records for analysis from which the 15 most frequently prescribed agents were analysed. This data set contained 1051 (90.0%) prescriptions (Table 1), prescribed for 645 (61.4%) males, 405 (38.5%) females and one (0.1%) other. The excluded 10.0% (n = 117) of prescriptions comprised 29 different antibacterial agents; each of which was prescribed ≤ 14 times (median 3). The top 15 agents were each prescribed, on average > 70 times (median 55).

**Table 1:** Frequency of prescribing of the top 15 antibacterial agent prescriptions on the annual day of the National Antimicrobial Prescribing Survey (NAPS) for the period 2013 to 2017

Antimicrobial agent	Individual year data N = 1051 (%)					Five year combined data N (%)
	2013	2014	2015	2016	2017	
Piperacillin-tazobactam	59 (24.4)	61 (21.0)	45 (28.1)	43 (27.4)	14 (6.9)	222 (21.1)
Cefazolin	36 (14.9)	41 (14.1)	30 (18.8)	20 (12.7)	38 (18.8)	165 (15.7)
Amoxicillin-clavulanic acid	24 (9.9)	26 (9.0)	16 (10.0)	12 (7.6)	15 (7.4)	93 (8.8)
Ceftriaxone	5 (2.1)	16 (5.5)	10 (6.3)	12 (7.6)	37 (18.3)	80 (7.6)
Metronidazole	15 (6.2)	21 (7.2)	4 (2.5)	12 (7.6)	26 (12.9)	78 (7.4)
Flucloxacillin	19 (7.9)	20 (6.9)	14 (8.8)	8 (5.1)	11 (5.4)	72 (6.9)
Vancomycin	15 (6.2)	17 (5.9)	5 (3.1)	8 (5.1)	12 (5.9)	57 (5.4)
Meropenem	17 (7.0)	14 (4.8)	8 (5.0)	7 (4.5)	9 (4.5)	55 (5.2)
Amoxicillin	12 (5.0)	17 (5.9)	5 (3.1)	8 (5.1)	6 (3.0)	48 (4.6)
Azithromycin	12 (5.0)	13 (4.5)	6 (3.8)	6 (3.8)	10 (5.0)	47 (4.5)
Cefalexin	6 (2.5)	9 (3.1)	5 (3.1)	8 (5.1)	7 (3.5)	35 (3.3)
Trimethoprim-sulfamethoxazole	8 (3.3)	12 (4.1)	3 (1.9)	4 (2.5)	4 (2.0)	31 (2.9)
Clindamycin	6 (2.5)	9 (3.1)	3 (1.9)	6 (3.8)	2 (1.0)	26 (2.5)
Ciprofloxacin	7 (2.9)	10 (3.4)	3 (1.9)	1 (0.6)	2 (1.0)	23 (2.2)
Doxycycline	1 (0.4)	4 (1.4)	3 (1.9)	2 (1.3)	9 (4.5)	19 (1.8)
TOTAL	242	290	160	157	202	1051

### Prevalence of antibacterial use

Prior to 2017, for most individual years, piperacillin-tazobactam was the most frequently prescribed antimicrobial agent (mean 25.2%; range 21.0–28.1%), falling to 6.9% in 2017 due to global stock shortages that year [15, 25]. Overall, cefazolin was the second most frequently prescribed agent except in 2017 when it was number one (18.8%) (Table 1).

As shown in Table 2 common indications included surgical prophylaxis (12.2%), community acquired pneumonia (CAP) (7.8%), skin and soft tissue infections (SSTI) (5.0%) and hospital acquired pneumonia (HAP) (3.8%).

**Table 2:** Comparison of external audit with internal assessment of hospital National Antimicrobial Prescribing Survey (NAPS) prescribing data for compliance with guidelines of individual agents, indications and audit years

		Compliance with guidelines									
	Total Number of Prescriptions n	Compliant with guidelines n (%)		Non-compliant with guidelines n (%)		Directed therapy n (%)		No guidelines available n (%)		Not assessable n (%)	
*Antimicrobial agent*		External	Internal	External	Internal	External	Internal	External	Internal	External	Internal
Piperacillin- tazobactam	222	43 (19.4)	73 (32.9)	116 (52.3)	91 (41.0)	37 (16.7)	25 (11.3)	12 (5.4)	29 (13.1)	14 (6.3)	4 (1.8)
Cefazolin	165	17 (10.3)	46 (27.9)	128 (77.6)	95 (57.6)	12 (7.3)	12 (7.3)	6 (3.6)	10 (6.1)	2 (1.2)	2 (1.2)
Amoxicillin-clavulanic acid	93	14 (15.1)	30 (32.3)	45 (48.4)	31 (33.3)	13 (14.0)	10 (10.8)	5 (5.4)	15 (16.1)	16 (17.2)	7 (7.5)
Ceftriaxone	80	39 (48.8)	42 (52.5)	31 (38.8)	22 (27.5)	4 (5.0)	8 (10.0)	0 (0.0)	5 (6.3)	6 (7.5)	3 (3.8)
Metronidazole	78	20 (25.6)	33 (42.3)	42 (53.8)	30 (38.5)	9 (11.5)	4 (5.1)	2 (2.6)	7 (9.0)	5 (6.4)	4 (5.1)
Flucloxacillin	72	31 (43.1)	36 (50.0)	24 (33.3)	14 (19.4)	15 (20.8)	18 (25.0)	2 (2.8)	3 (4.2)	0 (0.0)	1 (1.4)
Vancomycin	57	9 (15.8)	18 (31.6)	11 (19.3)	5 (8.8)	33 (57.9)	22 (38.6)	0 (0.0)	10 17.5)	4 (7.0)	2 (3.5)
Meropenem	55	4 (7.3)	12 (21.8)	14 (25.5)	7 (12.7)	32 (58.2)	19 (34.5)	1 (1.8)	16 (29.1)	4 (7.3)	1 (1.8)
Amoxicillin	48	9 (18.7)	18 (37.5)	27 (56.3)	13 (27.1)	9 (18.8)	10 (20.8)	0 (0.0)	6 (12.5)	3 (6.3)	1 (2.1)
Azithromycin	47	14 (29.8)	21 (44.7)	25 (53.2)	20 (42.6)	1 (2.1)	2 (4.3)	2 (4.3)	3 (6.4)	5 (10.6)	1 (2.1)
Cefalexin	35	5 (14.3)	5 (14.3)	28 (80.0)	19 (54.3)	0 (0.0)	8 (22.9)	0 (0.0)	3 (8.6)	2 (5.7)	0 (0.0)
Trimethoprim-sulfamethoxazole	31	12 (38.7)	19 (61.3)	15 (48.4)	3 (9.7)	3 (9.7)	2 (6.5)	1 (3.2)	7 (22.6)	0 (0.0)	0 (0.0)
Clindamycin	26	2 (7.7)	4 (15.4)	12 (46.2)	8 (30.8)	10 (38.5)	11 (42.3)	0 (0.0)	2 (7.7)	2 (7.7)	1 (3.8)
Ciprofloxacin	23	2 (8.7)	5 (21.7)	4 (17.4)	2 (8.7)	11 (47.8)	6 (26.1)	1 (4.3)	8 (34.8)	5 (21.7)	2 (8.7)
Doxycycline	19	5 (26.3)	7 (36.8)	11 (57.9)	7 (36.8)	2 (10.5)	3 (15.8)	0 (0.0)	1 (5.3)	1 (5.3)	1 (5.3)
Total	1051	226 (21.5)	369 (35.1)	533 (50.7)	367 (34.9)	191 (18.2)	160 (15.2)	32 (3.0)	125 (11.9)	69 (6.6)	30 (2.9)
*Indication*											
Surgical Prophylaxis	128	10 (7.8)	25 (19.5)	116 (90.6)	94 (73.4)	0 (0.0)	0 (0.0)	2 (1.6)	9 (7.0)	0 (0.0)	0 (0.0)
Community Acquired Pneumonia (CAP)	82	32 (39.0)	35 (42.7)	48 (58.5)	42 (51.2)	2 (2.4)	5 (6.1)	0 (0.0)	0 (0.0)	0 (0.0)	0 (0.0)
Skin and Soft Tissue Infections (SSTI)	53	17 (32.1)	24 (45.3)	28 (52.8)	18 (34.0)	8 (15.1)	6 (11.3)	0 (0.0)	5 (9.4)	0 (0.0)	0 (0.0)
Hospital Acquired Pneumonia (HAP)	40	11 (27.5)	24 (60.0)	27 (67.5)	10 (25.0)	1 (2.5)	1 (2.5)	0 (0.0)	4 (10.0)	1 (2.5)	1 (2.5)
Bacterial Osteomyelitis	38	5 (13.2)	10 (26.3)	9 (23.7)	4 (10.5)	24 (63.2)	19 (50.0)	0 (0.0)	5 (13.2)	0 (0.0)	0 (0.0)
Aspiration Pneumonia	38	16 (42.1)	22 (57.9)	17 (44.7)	12 (31.6)	4 (10.5)	2 (5.3)	0 (0.0)	0 (0.0)	1 (2.6)	2 (5.3)
Trauma (includes wounds)	36	7 (19.4)	9 (25.0)	18 (50.0)	13 (36.1)	11 (30.6)	6 (16.7)	0 (0.0)	8 (22.2)	0 (0.0)	0 (0.0)
Urinary Tract Infection	35	4 (11.4)	16 (45.7)	20 (57.1)	7 (20.0)	11 (31.4)	9 (25.7)	0 (0.0)	3 (8.6)	0 (0.0)	0 (0.0)
Annual audit results											
2013	242 (100.0)	52 (21.5)	85 (35.1)	124 (51.2)	89 (36.8)	33 (13.6)	0 (0.0)	16 (6.6)	61 (25.2)	17 (7.0)	7 (2.9)
2014	290 (100.0)	52 (17.9)	92 (31.7)	142 (49.0)	95 (32.8)	59 (20.3)	55 (19.0)	9 (3.1)	36 (12.4)	28 (9.7)	12 (4.1)
2015	160 (100.0)	28 (17.5)	54 (33.8)	97 (60.0)	64 (40.0)	25 (15.6)	26 (16.3)	1 (0.6)	12 (7.5)	9 (5.6)	4 (2.5)
2016	157 (100.0)	37 (23.6)	52 (33.1)	75 (47.8)	52 (33.1)	37 (23.6)	43 (27.4)	2 (1.3)	6 (3.8)	6 (3.8)	4 (2.5)
2017	202 (100.0)	57 (28.2)	86 (42.6)	95 (47.0)	67 (33.2)	37 (18.3)	36 (17.8)	4 (2.0)	10 (5.0)	9 (4.5)	3 (1.5)

### External Guideline Compliance Assessment

This external assessment found 50.7% (n = 533) prescriptions were non-compliant with any guidelines. Of 226 (21.6%) compliant prescriptions; 216 (20.6%) were compliant with Therapeutic Guidelines - Antibiotics and 10 prescriptions (1.0%) were compliant with hospital specific, WA-TAG or Australian Medicines Handbook guidelines (Table 2). One hundred and ninety one (18.2%) prescriptions were for therapy directed by an infectious diseases clinician or based on microbiological tests.

The highest rates of non-compliance were observed with cefalexin (28/35; 80.0%), cefazolin (128/165; 77.6%), piperacillin-tazobactam (116/222; 52.3%) and metronidazole (42/78; 53.8%). The highest rates of compliance with guidelines were seen for ceftriaxone (39/80; 48.8%) and flucloxacillin (31/72; 43.1%) (Table 2).

### External Appropriateness Assessment

Although more than half (551/1051; 52.4%) of antibacterial prescriptions were deemed appropriate (Table 3), overall, 39.7% (417) of prescriptions were inappropriate; including 44 (4.2%) prescriptions which were not required according to their recorded indication.

**Table 3:** Comparison of external audit with internal assessment of hospital National Antimicrobial Prescribing Survey (NAPS) prescribing data for appropriateness of individual agents, indications and audit years

	Appropriateness						
	Total Number of Prescriptions n (%)	Appropriate n (%)		Inappropriate n (%)		Not assessable n (%)	
*Antimicrobial agent*		External	Internal	External	Internal	External	Internal
Piperacillin-tazobactam	222 (21.1)	121 (54.5)	163 (73.4)	84 (37.8)	57 (25.7)	17 (7.7)	2 (0.9)
Cefazolin	165 (15.7)	54 (32.7)	100 (60.6)	107 (64.8)	64 (38.8)	4 (2.4)	1 (0.6)
Amoxicillin-clavulanic acid	93 (8.8)	42 (45.2)	59 (63.4)	33 (35.5)	30 (32.3)	18 (19.4)	4 (4.3)
Ceftriaxone	80 (7.6)	49 (61.3)	55 (68.8)	25 (31.3)	23 (28.7)	6 (7.5)	2 (2.5)
Metronidazole	78 (7.4)	40 (51.3)	48 (61.5)	33 (42.3)	28 (35.9)	5 (6.4)	2 (2.6)
Flucloxacillin	72 (6.9)	54 (75.0)	63 (87.5)	18 (25.0)	9 (12.5)	0 (0.0)	0 (0.0)
Vancomycin	57 (5.4)	41 (71.9)	51 (89.5)	12 (21.1)	6 (10.5)	4 (7.0)	0 (0.0)
Meropenem	55 (5.2)	40 (72.7)	52 (94.5)	7 (12.7)	2 (3.6)	8 (14.5)	1 (1.8)
Amoxicillin	48 (4.6)	23 (47.9)	33 (68.8)	23 (47.9)	14 29.2)	2 (4.2)	1 (2.1)
Azithromycin	47 (4.5)	21 (44.7)	30 (63.8)	19 (40.4)	16 (34.0)	7 (14.9)	1 (2.1)
Cefalexin	35 (3.3)	12 (34.3)	14 (40.0)	22 (62.9)	21 (60.0)	1 (2.9)	0 (0.0)
Trimethoprim-sulfamethoxazole	31 (2.9)	13 (41.9)	29 (93.5)	16 (51.6)	2 (6.5)	2 (6.5)	0 (0.0)
Clindamycin	26 (2.5)	17 (65.4)	19 (73.1)	7 (26.9)	7 (26.9)	2 (7.7)	0 (0.0)
Ciprofloxacin	23 (2.2)	14 (60.9)	18 (78.3)	4 (17.4)	4 (17.4)	5 (21.7)	1 (4.3)
Doxycycline	19 (1.8)	10 (52.6)	11 (57.9)	7 (36.8)	7 (36.8)	2 (10.5)	1 (5.1)
Total	1051 (100.0)	551 (52.4)	745 (70.9)	417 (39.7)	290 (27.6)	83 (7.9)	16 (1.5)
*Indication*							
Surgical Prophylaxis	128	17 (13.3)	48 (37.5)	111 (86.7)	80 (62.5)	0 (0.0)	0 (0.0)
Community Acquired Pneumonia (CAP)	82	46 (56.1)	50 (61.0)	36 (43.9)	32 (39.0)	0 (0.0)	0 (0.0)
Skin and Soft Tissue Infections (SSTI)	53	40 (75.5)	46 (86.8)	13 (24.5)	7 (13.2)	0 (0.0)	0 (0.0)
Hospital Acquired Pneumonia (HAP)	40	15 (37.5)	31 (77.5)	23 (57.5)	9 (22.5)	2 (5.0)	0 (0.0)
Bacterial Osteomyelitis	38	30 (78.9)	35 (92.1)	8 (21.1)	3 (7.9)	0 (0.0)	0 (0.0)
Aspiration Pneumonia	38	19 (50.0)	28 (73.7)	18 (47.4)	7 (18.4)	1 (2.6)	3 (7.9)
Trauma (includes wounds)	36	18 (50.0)	23 (63.9)	18 (50.0)	13 (36.1)	0 (0.0)	0 (0.0)
Urinary Tract Infection	35	24 (68.6)	28 (80.0)	11 (31.4)	7 (20.0)	0 (0.0)	0 (0.0)
Annual audit results							
2013	242 (100.0)	121 (50.0)	173 (71.5)	96 (39.7)	68 (28.1)	25 (10.3)	1 (0.4)
2014	290 (100.0)	142 (49.0)	202 (69.7)	115 (39.7)	78 (26.9)	33 (11.4)	10 (3.4)
2015	160 (100.0)	74 (46.3)	104 (65.0)	76 (47.5)	54 (33.8)	10 (6.3)	2 (1.3)
2016	157 (100.0)	93 (59.2)	121 (77.1)	57 (36.3)	33 (21.0)	7 (4.5)	3 (1.9)
2017	202 (100.0)	121 (59.9)	145 (71.8)	73 (36.1)	57 (28.2)	8 (4.0)	0 (0.0)

The most commonly prescribed antibacterial agent, piperacillin-tazobactam was inappropriately prescribed in 84/222 (37.8%) cases. Cefazolin was the most frequently deemed inappropriately prescribed antibacterial (107/165; 64.8%) (Table 3). Agents with high rates of appropriate prescribing included flucloxacillin (54/72; 75.0%), meropenem (40/55; 72.7%) and vancomycin (41/57; 71.7%); the latter two agents were also frequently prescribed as directed therapy at rates of 58.2% (32/55) and 57.9% (33/57), respectively.

### External versus internal findings

Overall, considerable non-concordance occurred between the external and internal assessments for compliance with guidelines (Table 2) and appropriateness (Table 3) of prescribing for all indications. Antibacterials were most frequently non-compliant for surgical prophylaxis according to this external study, with 116/128 (90.6%) compared with the internal hospital analysis 94/128 (73.4%). Similarly, external analysis could not replicate rates of non-compliant prescribing for other common indications including CAP, SSTI and HAP (Table 2). Analysis of the difference between external and internal audits revealed the largest discrepancy in compliance with guidelines to be in the 2015 audit (Table 2).

External assessment found 111/128 (86.7%) of surgical prophylaxis prescriptions were inappropriate, compared to 80/128 (62.5%) reported by internal analysis. Rates of inappropriate prescribing also differed between external and internal analyses for CAP, SSTI, and HAP (Table 3).

External findings could not replicate internal compliance and appropriateness assessments for the different antibacterial agents prescribed. External assessors found 116/222 (52.3%) of piperacillin-tazobactam and 128/165 (77.6%) of cefazolin prescriptions were non-compliant with guidelines; compared to 91/222 (41.0%) and 95/165 (57.6%) found by internal assessments respectively (Table 2). Inappropriate prescribing was reported by external assessment as 83/222 (37.4%) for piperacillin-tazobactam and 106/165 (64.2%) of cefazolin prescriptions; while internal assessment found lower rates of 57/222 (25.7%) and 64/165 (38.8%) respectively (Table 3).

More prescriptions were deemed ‘Not Assessable’ by external auditors for compliance (69/1051; 6.6%) (Table 2) and appropriateness (83/1051; 7.9%) (Table 3) parameters compared to internal assessors (30/1051; 2.9%) and (16/1051; 1.6%) respectively. This external audit reported fewer ‘No Guidelines Available’ assessments (32/1051; 3.0%) in the compliance parameter than internal assessors (125/1051; 11.9%) (Table 2).

In the assessment of the overall five year data, Cohen’s Kappa revealed a moderate agreement between internal and external prescription analyses for compliance with guidelines (0.49) and for appropriateness (0.50). Cross tabulations for Cohen’s Kappa analysis revealed that agreement between internal and external assessors varied for the different categories of compliance (Table 4) and appropriateness (Table 5). The largest discrepancy in agreement was in the “Compliant with guidelines” variable; as external assessors agreed with only 182/369 (49.3%) of internal assessments. There was higher agreement in the “Non-compliant with guidelines” variable with 323/367 (88.0%) of internal assignment being reproduced by external assessors. Overall, agreement between internal and external analyses was reached in 665/1051 (63.3%) of assessments for compliance with guidelines (Table 4). Non-compliant prescriptions due to an incorrect dose or frequency of administration were reported at rates of 208 (19.8%) prescriptions by external and 111 (10.6%) by internal assessors. Differences in the assessment of allergy 18 (1.7%) versus 11 (1.0%) and microbiology 36 (3.4%) versus 23 (2.2%) prescribing mismatch between external versus internal analysis were also respectively identified.

**Table 4:** Cohen’s Kappa cross tabulation for agreement of compliance with guidelines variable between internal and external analyses

			External Analysis				Total
		Compliant with guidelines	Non-compliant with guidelines	Directed therapy	No guidelines available	Not assessable	
Internal Analysis	Compliant with guidelines	182	145	28	5	9	369
	Non-compliant with guidelines	17	323	10	10	7	367
	Directed therapy	15	22	119	1	3	160
	No guidelines available	10	41	33	16	25	125
	Not assessable	2	2	1	0	25	30
Total		226	533	191	32	69	1051

**Table 5:** Cohen’s Kappa cross tabulation for agreement of appropriateness variable between internal and external analyses

	External Analysis				Total
		Appropriate	Inappropriate	Not assessable	
Internal Analysis	Appropriate	524	174	47	745
	Inappropriate	25	242	23	290
	Not assessable	2	1	13	16
Total		551	417	83	1051

Cohen’s Kappa cross tabulations of the appropriateness parameter (Table 5) revealed an overall agreement between internal and external auditors in 779/1051 (74.1%) of assessments. The largest discrepancy in agreement was in external auditors finding 174/745 (23.4%) of internally “appropriate” prescriptions to be “inappropriate”.

## Discussion

To the best of our knowledge, this is the first study undertaken in Australia to evaluate NAPS reliability by assessing concordance between hospital internal assessments and an external audit of antimicrobial prescribing.

Antibacterial agents prescribed in this hospital showed similar trends as national [26] and international [10, 11, 27] PPS. The most frequently prescribed antibacterial agents were penicillin-beta lactamase inhibitor combinations and first and third generation cephalosporins. Piperacillin-tazobactam prescribing (28.1%) was higher than national NAPS (6.3%)[28] and international PPS (7.7%)[10] data in 2015 and most other years. Higher overall rates of non-compliance (50.7%) than national NAPS (24.8%)[28] and international data [10] were identified. A global PPS of 53 countries reported rates of compliant prescribing of 77.4% [10].

Globally and in Australian hospitals, the main indications for antimicrobial prescriptions were pneumonia, SSTI and surgical prophylaxis [10, 29]. In this study, surgical prophylaxis accounted for 12.2% of antibacterial usage, which was higher than that reported in Canadian hospitals (8.9%)[11] and in Switzerland (9.1%)[27], but more closely aligned with the average five year NAPS rates (13.4%) in Australia [26, 28, 30–32]. This hospital reported a lower rate of antibacterial prescribing for CAP (7.8%) than national NAPS (11.4%) and Canadian data (14.4%)[11]. Similarly, antibacterial use for the treatment of HAP in this hospital (4.0%) was lower than 9.1% reported in Canada [11]. Pneumonia treatment was the most common indication (19.2%) in a global PPS of 53 countries [10].

Surgical prophylaxis retains one of the highest global rates of non-compliant prescribing with cefazolin being frequently used. It accounted for 64.1% of surgical prophylaxis prescriptions in this hospital, which was similar to rates in Canadian hospitals (69.7%) [11, 29]. Overall rates of non-compliant and inappropriate prescribing for this indication were 90.6% and 86.7% respectively, for this external evaluation, which differed significantly (p < 0.001) to internal assessments (73.4% and 62.5% respectively). International and Australian hospitals have reported inappropriate use of antibacterial agents for surgical prophylaxis due to treatment beyond one day; with rates ranging from 28.0 to 54.2% [11, 25, 27, 29]. In this study, only 18.0% of prescriptions for this indication were written for a single dose of prophylaxis, below 39.7% reported in other studies [11], but higher than 12.7% reported in Switzerland [27].

The guideline of a one day window of appropriate surgical prophylaxis prescribing is not unique to the Australian PPS interpretation [11, 33]. However, NAPS indicates this 24 h period is valid where there are no guidelines that direct therapy otherwise. Rather than a single pre-surgical, prophylactic dose, as recommended by the Therapeutic Guidelines - Antibiotics [16], most prescriptions were written for ongoing dosing and thus considered non-compliant by this external assessment. This accounted for most of the discrepancy between the external and internal evaluations for this indication. Internal auditors deemed prescriptions within the 24 h window to be appropriate, regardless of the guidelines. Documentation of surgical incision and antibacterial administration timing, as well as post-surgical dose requirements may improve reproducibility [29].

The average five year rates of non-compliance with guidelines was higher in this hospital whether by external (50.7%) or internal (34.9%) assessments, compared to the national data (24.8%). The largest discrepancy in non-compliance assessment between external (60.0%) and internal (40.0%) audits was reported in 2015. This was largely due to the update (in August 2015) of the Therapeutic Guidelines - Antibiotics, which changed commonly prescribed antibacterial agents dosage and frequency of administration regimens, but these were not reflected in the internal assessments carried out in November. Inappropriate prescribing was reported similarly as lower rates by internal (27.6%) than external (39.7%) assessments. These were both higher than national rates (22.5%), while international rates ranged between 14.1% and 78.9% [34].

It can be expected that individual hospital NAPS assessments will vary from the mean pooled national data to some degree, due to geographical variations affecting differences in antibacterial stock availability. Other factors include local hospital guidelines, resistance patterns, private and public sector policy differences and remoteness level of the institution from major cities [10, 34]. Similar concerns are expressed in a comparison of international PPS data and their reflection of global antibacterial usage [34]. The hospital in this study was a public hospital in a major city; while NAPS data pools national public and private sector, city, remote and regional hospitals data. Chi square tests indicated that the profile of both compliance and appropriateness differed significantly between internal and external assessments (p < 0.001). Exploring potential reasons for this difference may improve assessment reproducibility. While acknowledging that this audit of a single hospital limits generalisability, a much larger number of hospitals would require investigation to establish findings that would be generalisable. This study is not representative of national NAPS, it does however warrant further, large scale investigations into the need to validate this PPS as an AMS audit tool. Several limitations, including access to only the prescribing data pre-collected by hospital staff, have impacted the scope of this research.

The retrospective design further limited data analysis of 2016 results since the date of prescribing was not recorded, thus appropriateness of duration of therapy was not assessable. Insufficient reporting and missing information on indications and microbiological test results could influence the external audit increasing the level of non-assessable reports. Development of standardised data collection which fills the gaps and justifies decision making is a necessary inclusion to improve NAPS data reproducibility.

It is unclear from the available data why ‘no guidelines available’ was reported at a higher rate by internal assessors compared to external assessors (11.9% versus 3.0%) which again reinforces the need for additional comments boxes to explain decisions. To improve reproducibility of PPS results, we recommend inter- and intra-rater reliability checks be carried out every year ahead of NAPS. These reliability checks are among the most common initiatives to enhance reproducibility of antimicrobial use data [33, 35].

## Conclusion

High rates of non-compliant and inappropriate prescribing were identified in this hospital, consistent with national and international concerns regarding antibacterial agent prescribing. This external audit was unable to replicate internal hospital assessments for the studied parameters. NAPS and other international PPS should provide sufficient robustness to enable acceptably reproducible data to be obtained to reflect hospital antimicrobial prescribing practice; especially since it is an AMS assessment tool for many hospitals. Further research into the reproducibility of NAPS results and exploration of the completeness of documentation of data used in evaluating appropriateness is desirable.

## Electronic supplementary material

Below is the link to the electronic supplementary material.

Supplementary Material 1
