# Supplementary material for: A Retrospective, Longitudinal External Study of the robustness and reproducibility of National Antibacterial Prescribing Survey Data
Source: Int J Clin Pharm. 2022 Jun 6;44(4):956–65. doi: 10.1007/s11096-022-01411-w (PMC9393140; doi:10.1007/s11096-022-01411-w)

National Antimicrobial Prescribing Survey data collection form template. [15]


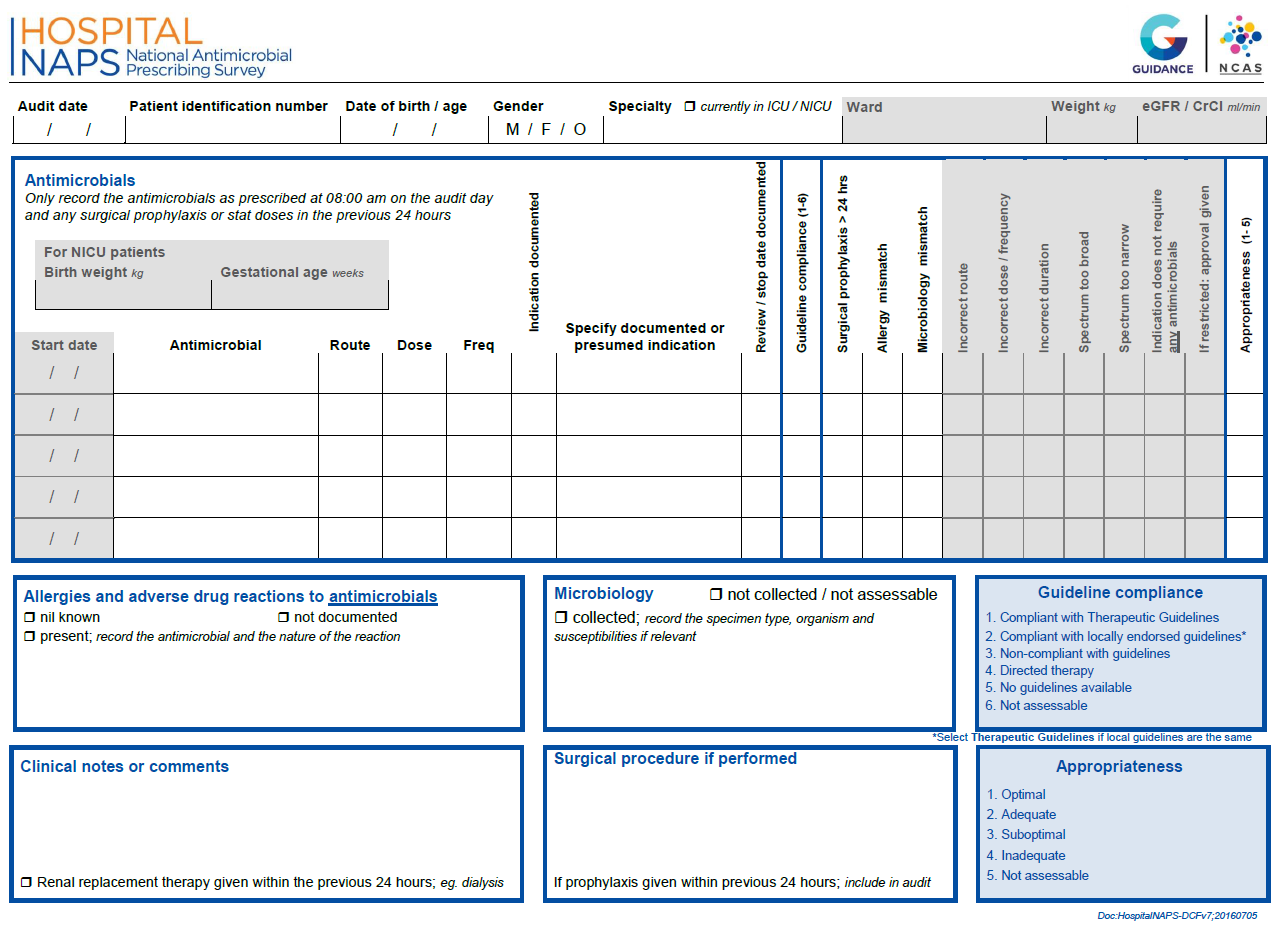
National Antimicrobial Prescribing Survey appropriateness assessment guidelines [15].


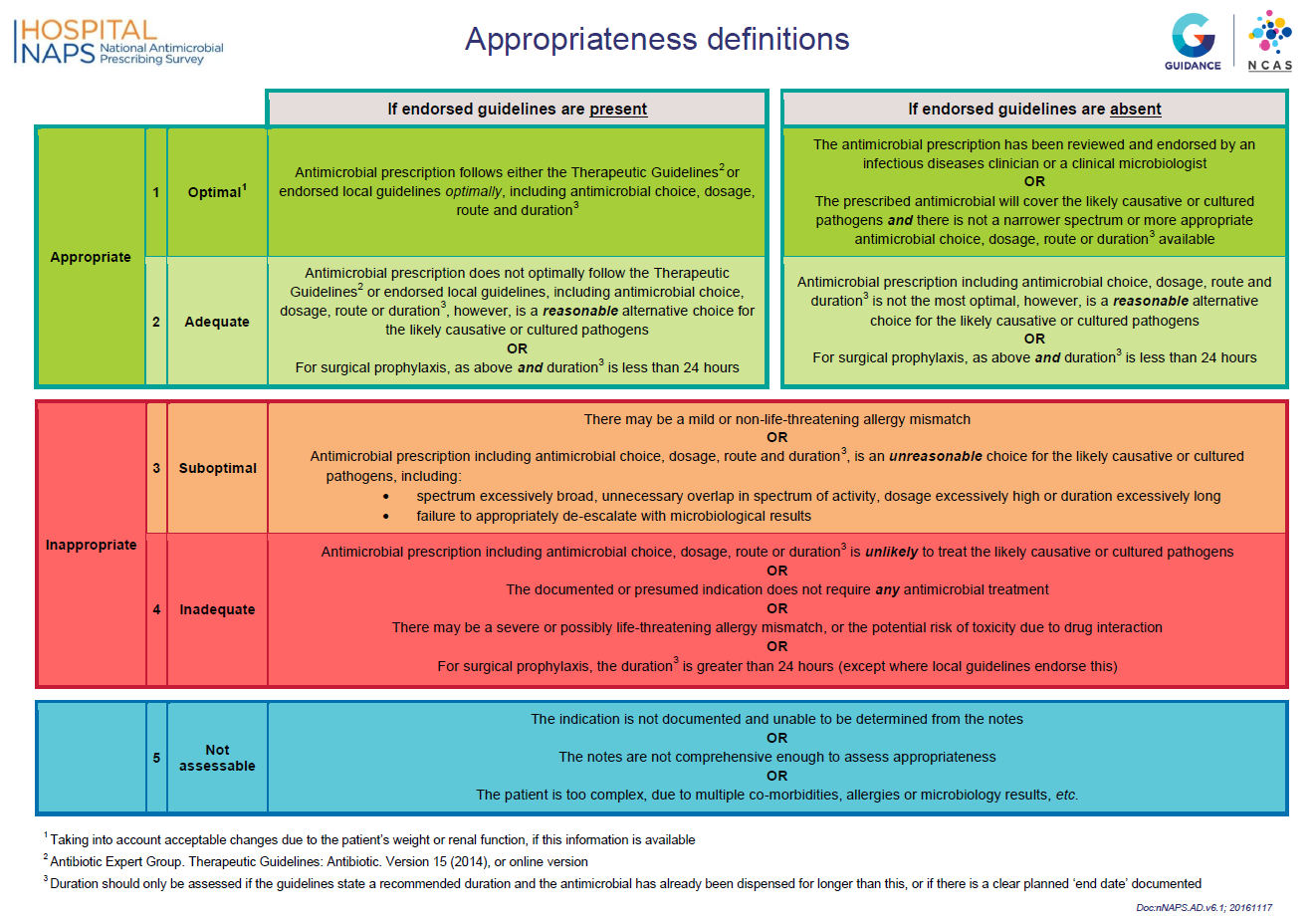

Supplement: Supplementary file 1 — Supplementary Material 1 [file 11096_2022_1411_MOESM1_ESM.docx]
